# Supplementary material for: Molecular and Clinicopathological Characterization of a Prognostic Immune Gene Signature Associated With MGMT Methylation in Glioblastoma
Source: Front Cell Dev Biol. 2021 Feb 5;9:600506. doi: 10.3389/fcell.2021.600506 (PMC7892978; doi:10.3389/fcell.2021.600506)
Supplement: Supplementary file 1 [file Data_Sheet_1.ZIP › Supplementary_Figures.DOCX]

**Supplementary figures**


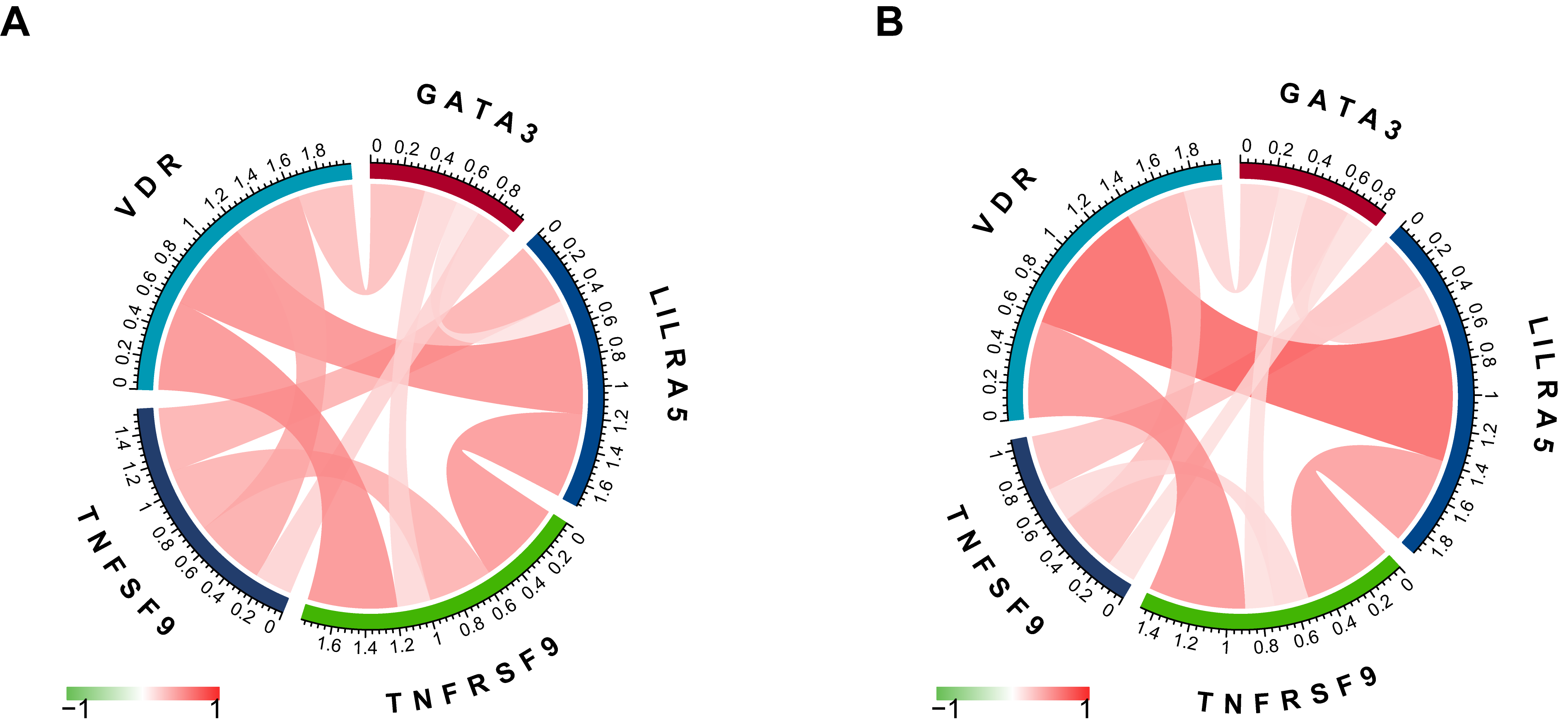


**Figure S1.** Correlations between the five prognostic immune genes in the TCGA (**A**) and CGGA RNA-seq (**B**) datasets.


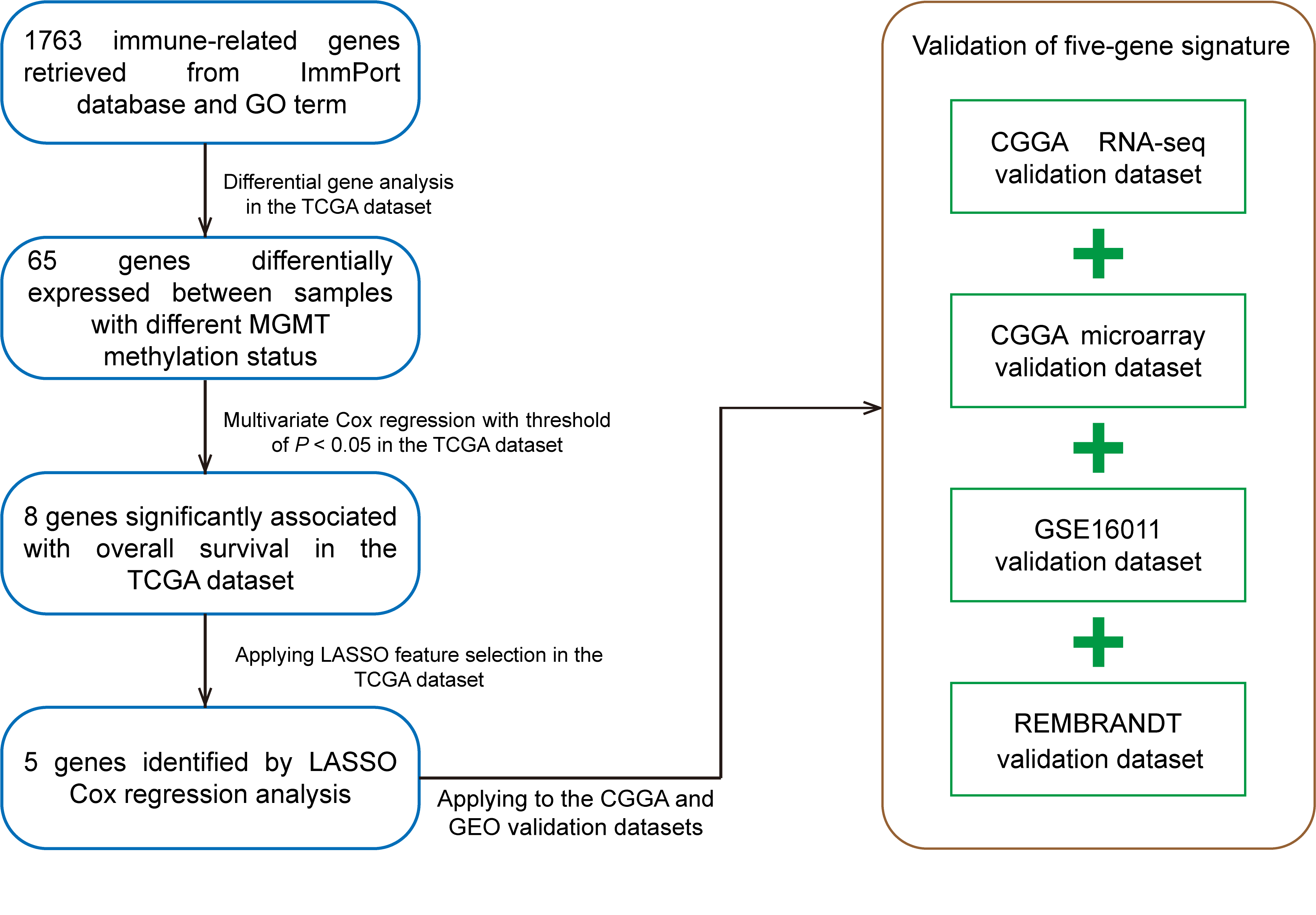


**Figure S2.** A schematic view of MGMT methylation related immune gene selection and prognostic gene signature development.


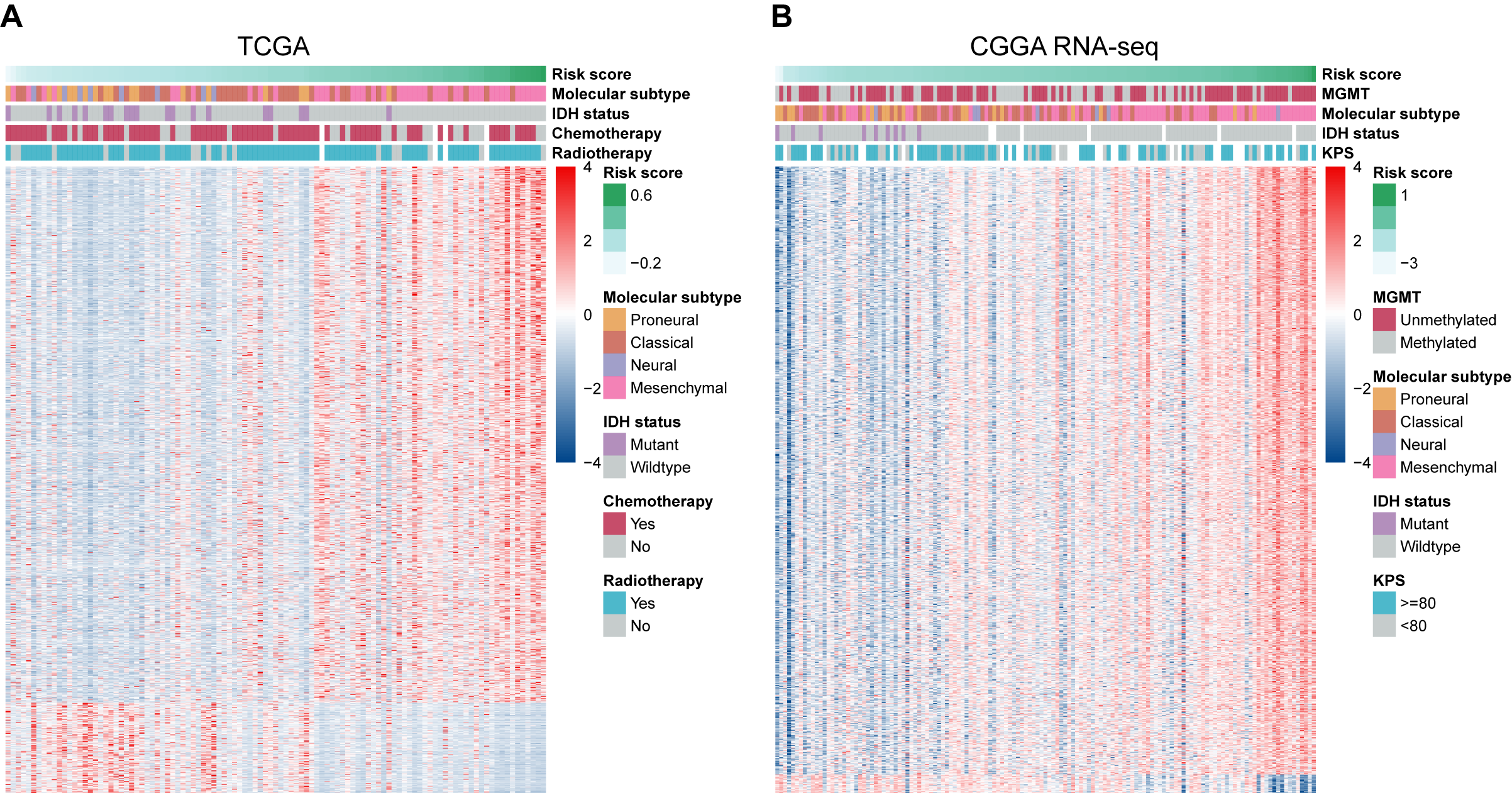


**Figure S3.** Genes strongly associated with risk score in both the TCGA (**A**) and CGGA RNA-seq (**B**) cohorts. The correlation coefficients were obtained from Pearson correlation analyses.


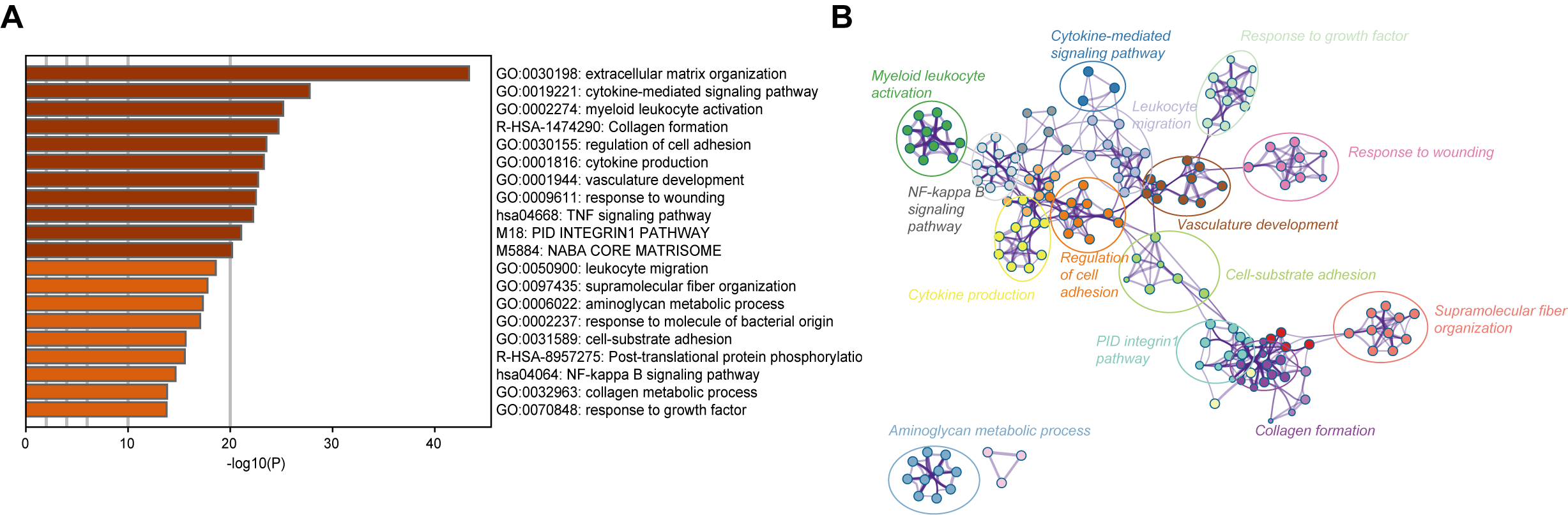


**Figure S4.** Biological functions of the risk signature in the CGGA RNA-seq dataset. **A.** Bar plot showing the top 20 terms derived from the gene set enrichment of risk score. The x-axis represents statistical significance. **B.** The enrichment network plot visualizing the relationship between a set of representative terms. Each term is assigned with a unique color.


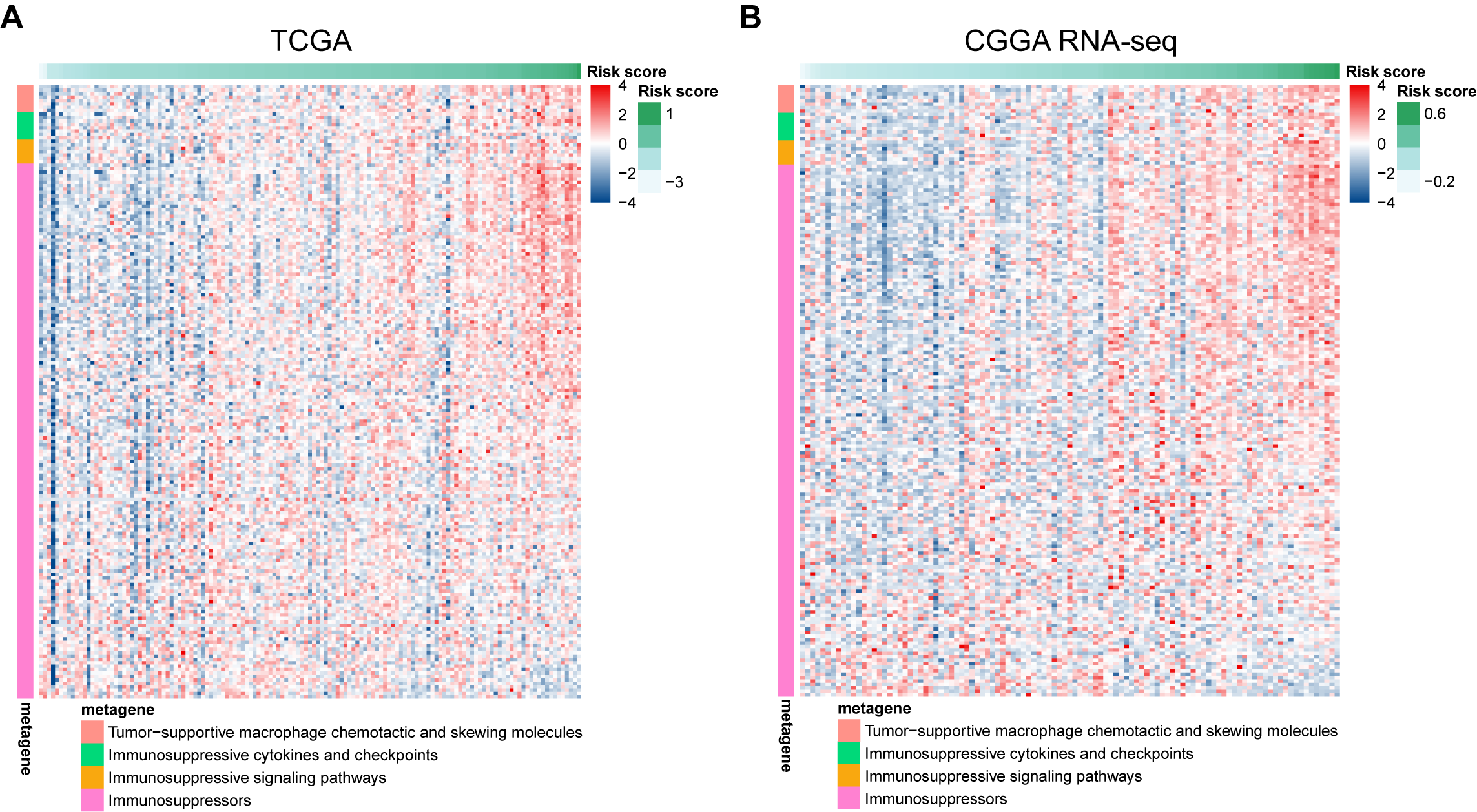


**Figure S5.** The risk signature is associated with immunosuppression in GBM samples. Heatmaps illustrating the association between risk score and glioma-associated immunosuppressive activities in the TCGA (**A**) and CGGA RNA-seq (**B**) cohorts.


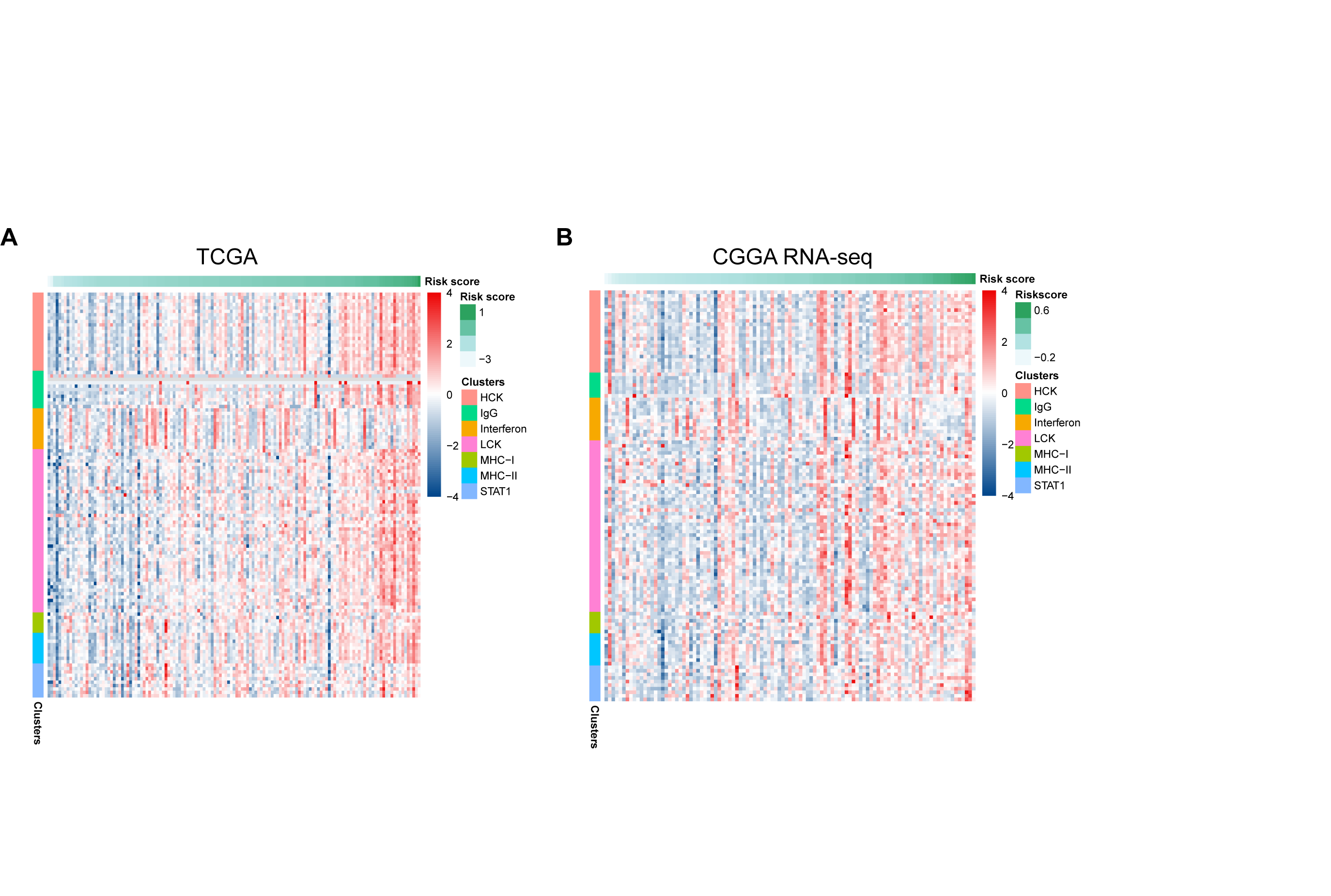


**Figure S6.** The risk signature is associated with the inflammatory response in GBM samples. Heatmaps illustrating the the association between risk score and predicted inﬂammatory activities in GBM samples from the TCGA (**A**) and CGGA RNA-seq (**B**) cohorts.


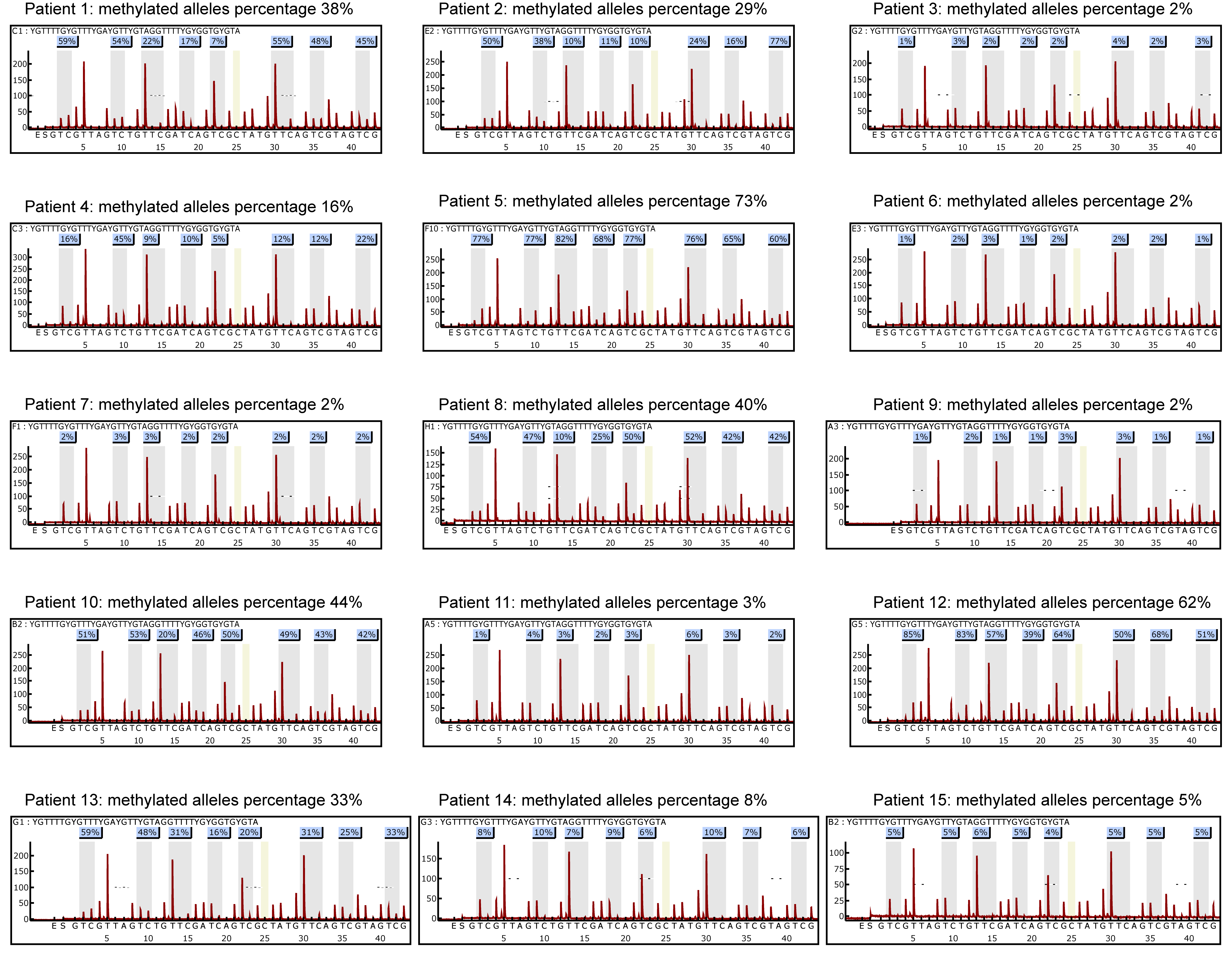


**Figure S7.** PSQ results of *MGMT* promoter in 15 GBM samples. The average methylated alleles percentage over 10% represents *MGMT* methylation.


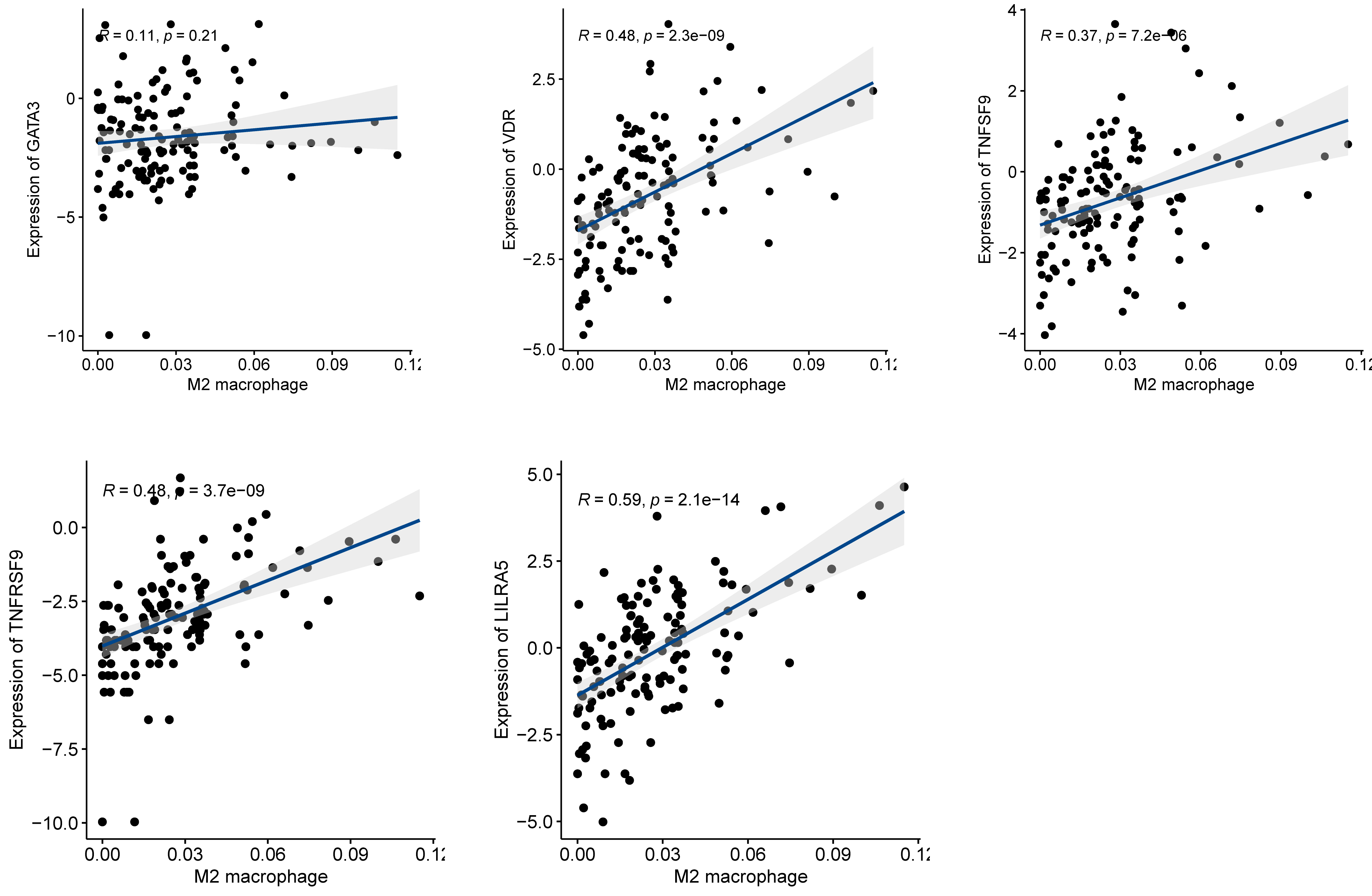


**Figure S8.** The correlation between expression levels of five key genes (*GATA3*, *VDR*, *TNFSF9*, *TNFRSF9*, and *LILRA5*) and the estimated M2 macrophage scores in the TCGA-GBM dataset. The coefficient and P-value were obtained from Pearson correlation analysis.


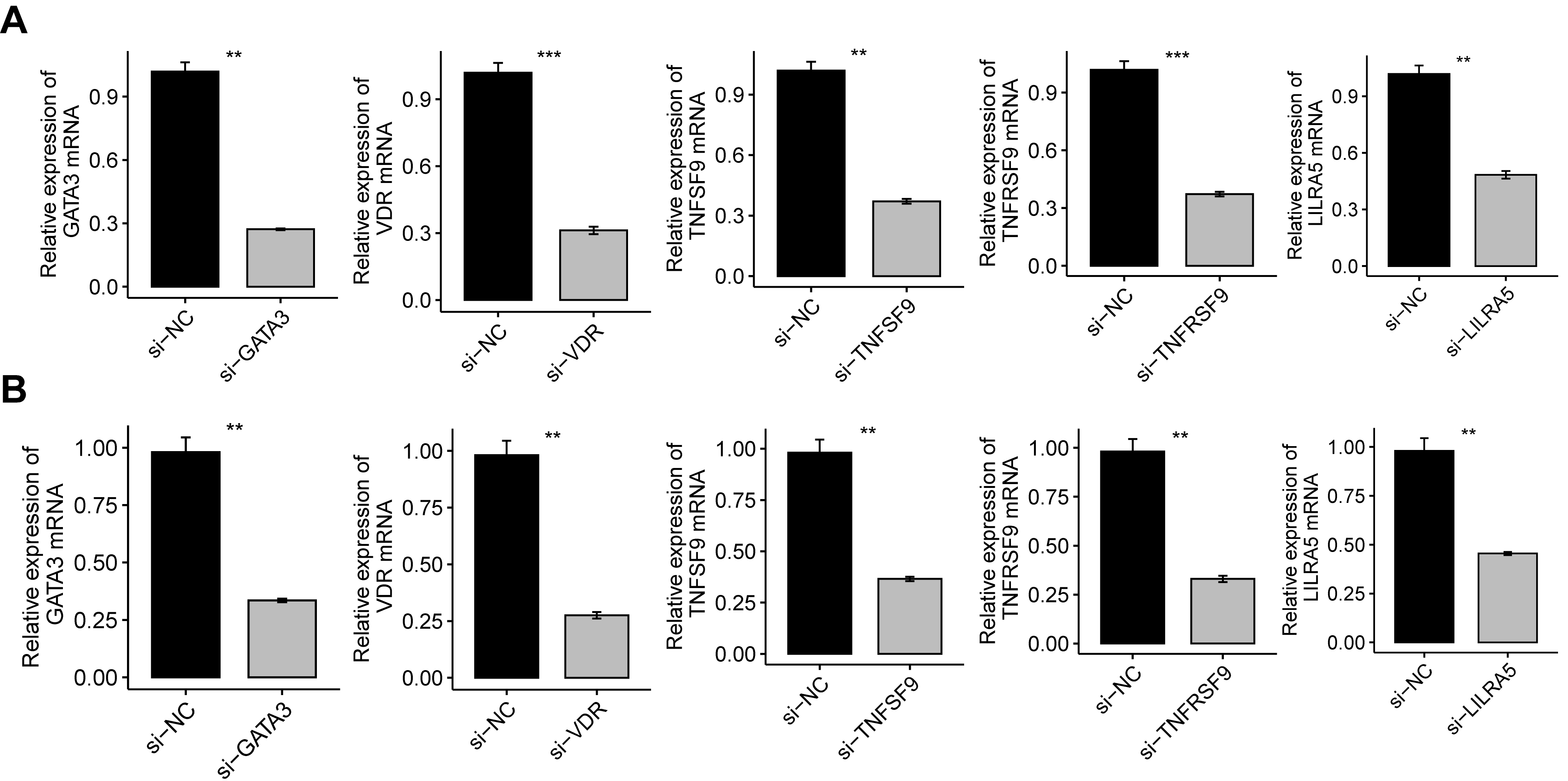


**Figure S9.** The silencing efficiency of the siRNAs for the five genes (*GATA3*, *VDR*, *TNFSF9*, *TNFRSF9*, and *LILRA5*) was confirmed in both U87 (**A**) and U251 (**B**) cells using qRT-PCR.
