# Supplementary material for: Molecular and Clinicopathological Characterization of a Prognostic Immune Gene Signature Associated With MGMT Methylation in Glioblastoma
Source: Front Cell Dev Biol. 2021 Feb 5;9:600506. doi: 10.3389/fcell.2021.600506 (PMC7892978; doi:10.3389/fcell.2021.600506)
Supplement: Supplementary file 1 [file Data_Sheet_1.ZIP › Table_S1.DOCX]

| **GBM datasets** | **Number of samples** | **Platforms** | **Link** |
| --- | --- | --- | --- |
| TCGA | 165 | Illumina HiSeq | https://portal.gdc.cancer.gov/ |
| CGGA RNA-seq | 113 | Illumina HiSeq | http://www.cgga.org.cn/download.jsp |
| CGGA microarray | 112 | Agilent Whole Human Genome Array | http://www.cgga.org.cn/download.jsp |
| GSE16011 | 159 | Affymetrix GeneChip Human Genome U133 Plus 2.0 Array | https://www.ncbi.nlm.nih.gov/geo/query/acc.cgi?acc=GSE16011 |
| REMBRANDT | 220 | Affymetrix Human Genome U133 Plus 2.0 Array | https://www.ncbi.nlm.nih.gov/geo/query/acc.cgi?acc=GSE108474 |

**Table S1. The detailed information about five GBM datasets used in the study.**
